# Dual Photoluminescence in Low-Temperature Phase of CsSnI_3_ Nanocrystals

**DOI:** 10.1021/jacs.5c10595

**Published:** 2025-08-06

**Authors:** Kyle T. Kluherz, Jacob L. Shelton, Nicholas J. Weadock, Noemi Leick, Peter C. Sercel, Matthew C. Beard

**Affiliations:** † 53405 National Renewable Energy Laboratory, Golden, Colorado 80401, United States; ‡ University of Colorado Boulder, Boulder, Colorado 80303, United States; § Center for Hybrid Organic Inorganic Semiconductors for Energy, Golden, Colorado 80401, United States

## Abstract

The expression of metal lone-pair electrons is hypothesized to underpin many of the interesting properties of inorganic halide perovskite semiconductors. Recently, a stable low-temperature monoclinic polar phase was predicted for CsSnBr_3_ and CsSnI_3_, opening the possibility of direct investigation of a ferroelectric distorted structure compared to the undistorted structure. To date, there have been no experimental reports of such a structure in CsSnI_3_, and the low-temperature optical properties of CsSnI_3_ nanocrystals have remained unexplored. Here we report optical and structural evidence of a phase transition around 240 K in 8.9 nm CsSnI_3_ nanocrystals. Several changes in optical behavior occur below this transition point, including high-energy photoluminescence (PL) that emits concurrently with the exciton PL. The emergence of this high-energy PL is correlated with X-ray diffraction (XRD) and differential scanning calorimetry (DSC) supporting a phase transition from the orthorhombic structure between 240–200 K. Transient absorption measurements show an increase in the excited state lifetimes, i.e., slowed carrier cooling, at 200 K when photoexciting with photon energies above the high-energy state, consistent with slowed carrier cooling and emergence of high-energy PL. We hypothesize that the slowed carrier cooling is distinctive to this phase transition that modifies both the electronic and phonon structures that dictate excited-state carrier dynamics, and we discuss these changes.

## Graphical abstract

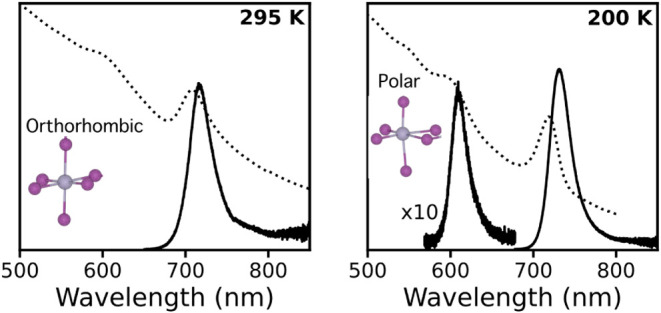

## Introduction

The halide perovskites are of great interest for their excellent optoelectronic properties, including such novel properties as defect tolerance,^1–3^ bright ground states, Rashba effects,^4–7^ and slowed hot carrier cooling.^8–10^ Of particular interest for cutting-edge solar energy conversion, several studies have reported pronounced slowed hot carrier cooling and hot carrier photoluminescence (PL) in the tin iodide perovskites.^9,11–15^ The hot carrier buildup in these systems has been attributed to a hot-phonon bottleneck effect, which is magnified by changes in optical phonon frequencies in the tin halide perovskite semiconductors as compared with the lead halide perovskites.^13^ However, these prolonged hot carrier lifetimes have all been reported under large pump fluences, and the hot carrier PL has only been observed as a high-energy tail or asymmetric broadening toward the blueside of the exciton PL line shape. To date, no work has considered how changes to the structure, such as a recently discovered ferroelectric phase,^16^ may alter these unique properties of the tin iodide perovskites. Additionally, several improved synthesis methods for the preparation of tin perovskite nanocrystals (NCs) have been recently reported, with improved optical properties, including PLQY of up to 18.4%^17^ and containing a clear exciton absorption peak, in contrast to previously reported Sn perovskite NCs with fairly featureless absorption spectra.^18–21^

Despite this interest in tin halide perovskite semiconductors, there are very few reports of their low-temperature properties. Several reports exist on the low-temperature optical properties of their lead halide counterparts, which exhibit decreasing bandgaps and decreasing radiative lifetimes with decreasing temperature in the NCs.^22–26^ Two reports found similar behavior in MASnI_3_ and CsSnI_3_ thin films, with intrinsic optical properties comparable to the lead halide perovskites.^27,28^ In contrast, splitting of the PL peaks was observed in CsSnBr_3_ microplates below 70 K, but not in CsSnI_3_,^29^ suggesting a transformation in optical properties tied to the halide species. Later, a phase transition of bulk (polycrystalline and single-crystal) CsSnBr_3_ to a polar, ferroelectric P21 structure was observed in this exact temperature range.^16^ A pair distribution function study found evidence of both a monoclinic low-temperature phase and Sn–Br–Sn bond distortions in CH_3_NH_3_SnBr_3_ alloyed with CsSnBr_3_,^30^ suggesting local symmetry breaking around the tin sites. Theoretical calculations further predict a stable polar phase for both CsSnI_3_ and CsSnBr_3_, with the possibility of a static Rashba effect resulting from the structural expression of the lone pair s electrons on the Sn site.^6^ However, to date, there are no reports of the optical properties of these predicted phases, and the iodide polar phase remains undiscovered.

In this work, we investigated the low-temperature optical properties of CsSnI_3_ NCs and correlated them with an observed phase change in temperature-dependent XRD measurements. We found an initial linear dependence of the optical bandgap on temperature similar to that observed for the lead halide perovskites,^22^ which ends around 240 K before entering a new linear regime below 230 K. Additionally, a new high-energy peak appears in the PL spectra, which increases relative to the exciton emission intensity. Notably, this behavior is distinct from the previously observed hot carrier emission in tin halide perovskites. We conclude that these novel optical properties are the result of a newly observed phase transition in CsSnI_3_, possibly the predicted low-temperature polar phase. We investigated the temperature-dependent carrier dynamics using transient absorption spectroscopy and found an increase in the excited-state lifetimes of the high-energy state that is correlated to the observed high-energy PL. We propose a reduction in carrier-phonon coupling and changes to the excited-state electronic structure indicative of the polar phase as the source of slowed hot carrier cooling, allowing for the emergence of the high-energy PL.

## Results

We synthesized CsSnI_3_ NCs according to the method reported by Gahlot et al.^18^ (see Experimental for further details), chosen for the distinct exciton absorption it yields. Figure 1a shows the steady-state absorption and PL of the sample with a sharp exciton absorption feature at 713 nm and band edge emission with a Stokes shift of 8 nm. The long tail toward the lower energy observed in the optical density and corresponding asymmetric PL line shape (Figure S1) is believed to be primarily the result of light scattering resulting from particle agglomeration in solution, and these features were reduced by suspending the NCs in a polystyrene matrix. Figure 1b shows high-energy XRD (heXRD) data of a film of CsSnI_3_ NCs along with a reference pattern and labeled peak indices. The pattern indexes cleanly to the reference structure of orthorhombic (Pnam) CsSnI_3_ (ICSD# 69996), with no evidence of any additional phases or peaks, indicating a single-phase product. TEM characterization (Figure 1c–e) exhibits a highly monodisperse population of NCs averaging 8.9 nm edge length with a 1 nm standard deviation (Figure 1c inset) and sharp electron diffraction pattern, which also indexes cleanly to the orthorhombic CsSnI_3_ structure (Figure S2), with possible evidence of a preferential orientation along the <100> axis on the TEM grid.

**Figure 1:**
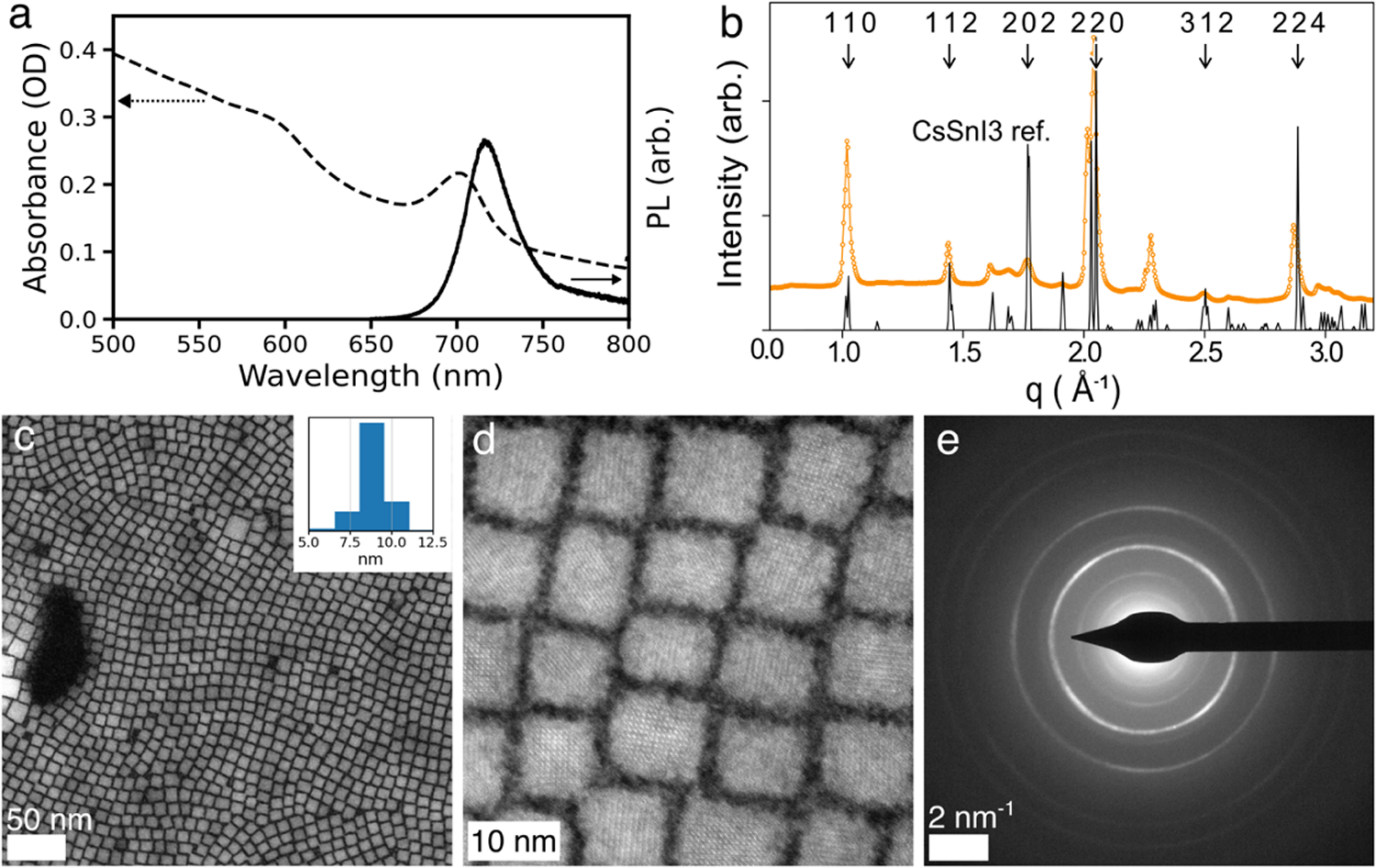
Characterization of CsSnI_3_ nanocrystals at 298 K, synthesized via hot injection. (a) Absorbance and photoluminescence (excitation wavelength: 600 nm). (b) XRD pattern of nanocrystal film with select peaks from bulk CsSnI_3_ labeled. (c, d) Representative STEM images. Inset in panel (c) shows a size distribution histogram. (e) Electron diffraction pattern.

We proceeded to measure the low-temperature optical properties of CsSnI_3_ NCs, both as an NC film and dispersed in a polystyrene matrix. Figure 2 shows the temperature-dependent absorbance and PL of CsSnI_3_ NCs for both cooling and heating cycles. In the absorbance data (a), we can observe two features, the exciton peak at lower energies and a higher energy peak around 600 nm, both of which gradually red-shift upon cooling, similar to behavior observed in other halide perovskite systems.^22,31^ The remainder of the features, at higher energies in the spectra, appear to remain unchanged in position and simply decrease in intensity upon cooling, although it is possible that the breadth of the absorption features could be eclipsing minor changes in position. A similar red shift is observed for the exciton PL (b), starting at 713 nm (1.734 eV) at 298 K and gradually moving to 737 nm (1.681 eV) at 180 K while simultaneously increasing in intensity. However, unlike in previously reported halide perovskite literature,^22,24,28,31^ the temperature-dependence of the exciton emission energy is not a single linear trend. A clear change in both the absorption and PL peak energy trends (c, d) can be observed between 250 and 230 K, which is reproducible on heating the sample back to 298 K. The absorption exhibits two linear regimes, one between 298 and 250 K (marked by a dashed line), the other from 230 K proceeding all the way down to 80 K, with a flat region from 250–230 K. The exciton PL exhibits similar behavior, although instead of a brief plateau between the two regimes, the energy jumps by about 10 meV. Additionally, a second, high-energy (HE) peak in the PL begins to emerge within this temperature range, and gradually grows in intensity relative to the exciton peak (Figure S10c). Figure 2d plots the positions of both PL peaks as a function of temperature. The HE peak disappears above 240 K in the PL data. This behavior was found to be reproducible across heating and cooling cycles and between samples. A similar heating trend to the absorbance data was found in the PL data (Figure S9). Additionally, the ratio of integrated peak intensities between the HE peak and the exciton peak (Figure S10) was found to increase with decreasing temperature, suggesting the HE emission is becoming more competitive at lower temperatures.

**Figure 2:**
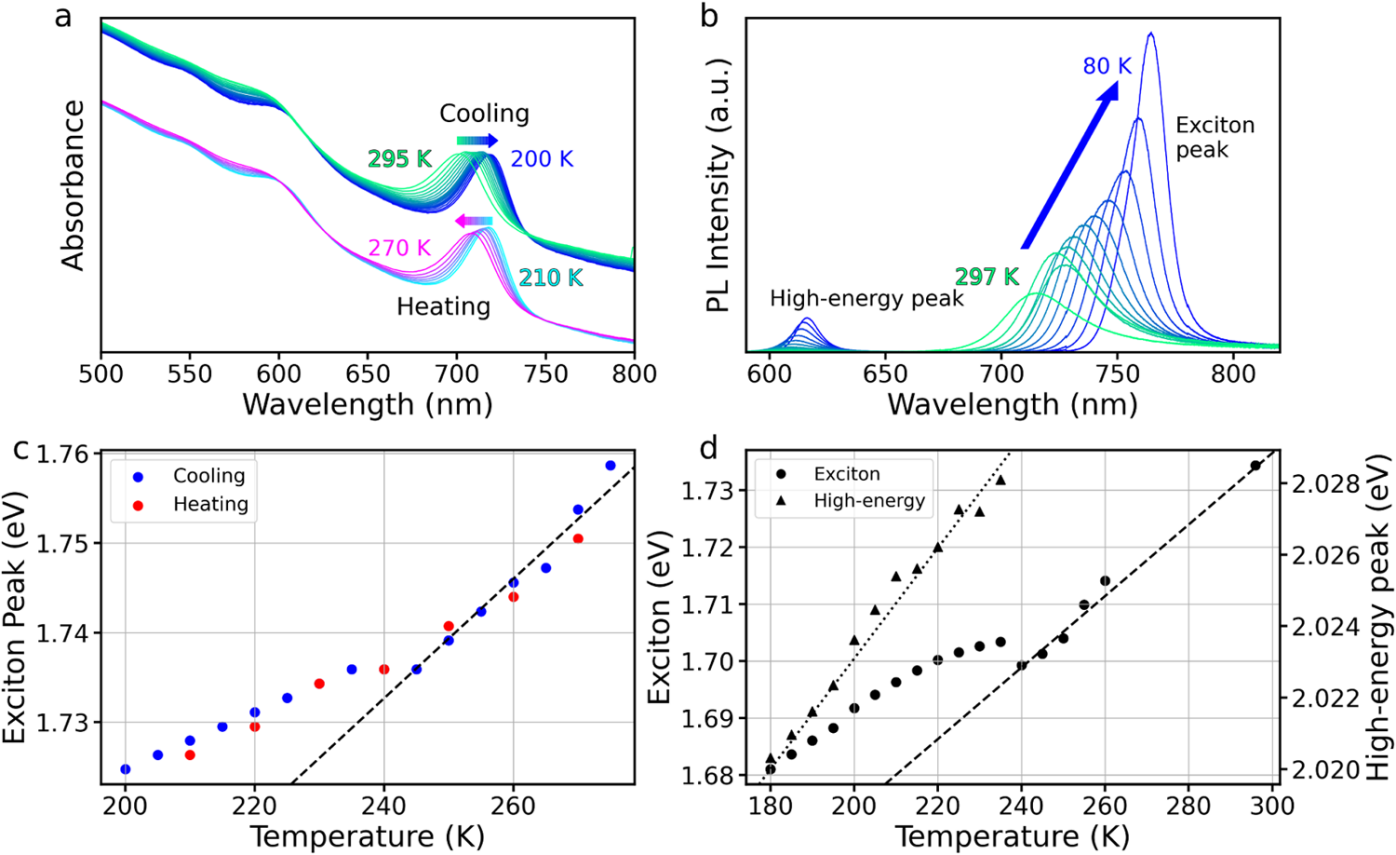
Temperature-dependent optical properties of CsSnI_3_ NCs. (a) Cooling and heating absorption spectra of CsSnI_3_ NC film from 295–200 K. (b) Low-temperature PL spectra of CsSnI_3_ NC films, from 297–80 K. (c) Absorption energy of exciton peak measured as a function of sample temperature for the cooling–heating cycle. The dashed line serves as a guide to the eye for trend from 275–245 K. (d) PL peak energies as a function of temperature for exciton emission and a new high-energy peak which appears below 240 K. The dashed line serves as a guide to the eye for the trend from 295–240 K, while the dotted line shows the trend in the high-energy peak.

To determine whether this optical behavior was related to a possible structural phase transition, we performed a low-temperature structural characterization. Figure 3 shows temperature-dependent XRD data, differential scanning calorimetry (DSC) data, and the octahedral network for orthorhombic and polar structure models of CsSnI_3_ NCs. The XRD patterns (a, b), shown in q-space, match cleanly to the reference pattern for CsSnI_3_ (ICSD# 69996) at 298 K, with no evidence of peaks from the oxidized phase, Cs_2_SnI_6_ (ICSD# 760462), at any temperature. The primary reflections are retained across temperatures, with a slight contraction of the lattice upon cooling. Additionally, several subtle changes were observed upon cooling, as highlighted in Figure 3b. A new peak appears at 1.56 Å^–1^ adjacent to the (112) peak, the loss of the distinct (120) and (013) peaks at 1.66 and 1.71 Å^–1^, respectively, suggests the appearance of additional reflections, the (202) peak at 1.79 Å^–1^ decreases in intensity, and the (004) peak at 2.05 Å^–1^ decreases into a shoulder. The heat flow extracted from DSC (c) shows several features indicative of progressive phase transitions between 270 and 220 K. While the sharp peak ∼260 K is likely attributed to the first phase transition of the orthorhombic to a possible monoclinic structure,^6^ the shallower peaks between 250 and 220 K are consistent with phase changes associated with polar crystal reorientation, requiring weaker energy inputs/outputs.^16^ Taken together, these changes indicate a phase change in the sample to slightly lower symmetry while maintaining the majority of the perovskite structure. This is consistent with what would be expected for a transition to the polar phase, and the XRD pattern of the predicted polar phase,^6^ calculated for 9 nm nanocrystals, is shown in Figure 3b. The first three changes described above correspond well with the calculated phase, but the positions of the (004) and (220) peaks do not seem to match that predicted by Swift and Lyons.^6^ This could suggest the polar phase has slightly different unit cell dimensions than those predicted (although no changes in other reflections are seen) or that the full polar transition happens at a lower temperature than was observed here. Two polar phases, with different transition temperatures, have been observed in bulk CsSnBr_3_ ^16^ and MASnBr_3_,^32^ with only one of the phases definitively identified as ferroelectric; thus, similar behavior is likely in the CsSnI_3_. This conclusion is supported by the multiple thermal events observed in the DSC heat flow trends (Figure 3c). Further structural characterization is needed to elucidate the exact structure of this new phase.

**Figure 3:**
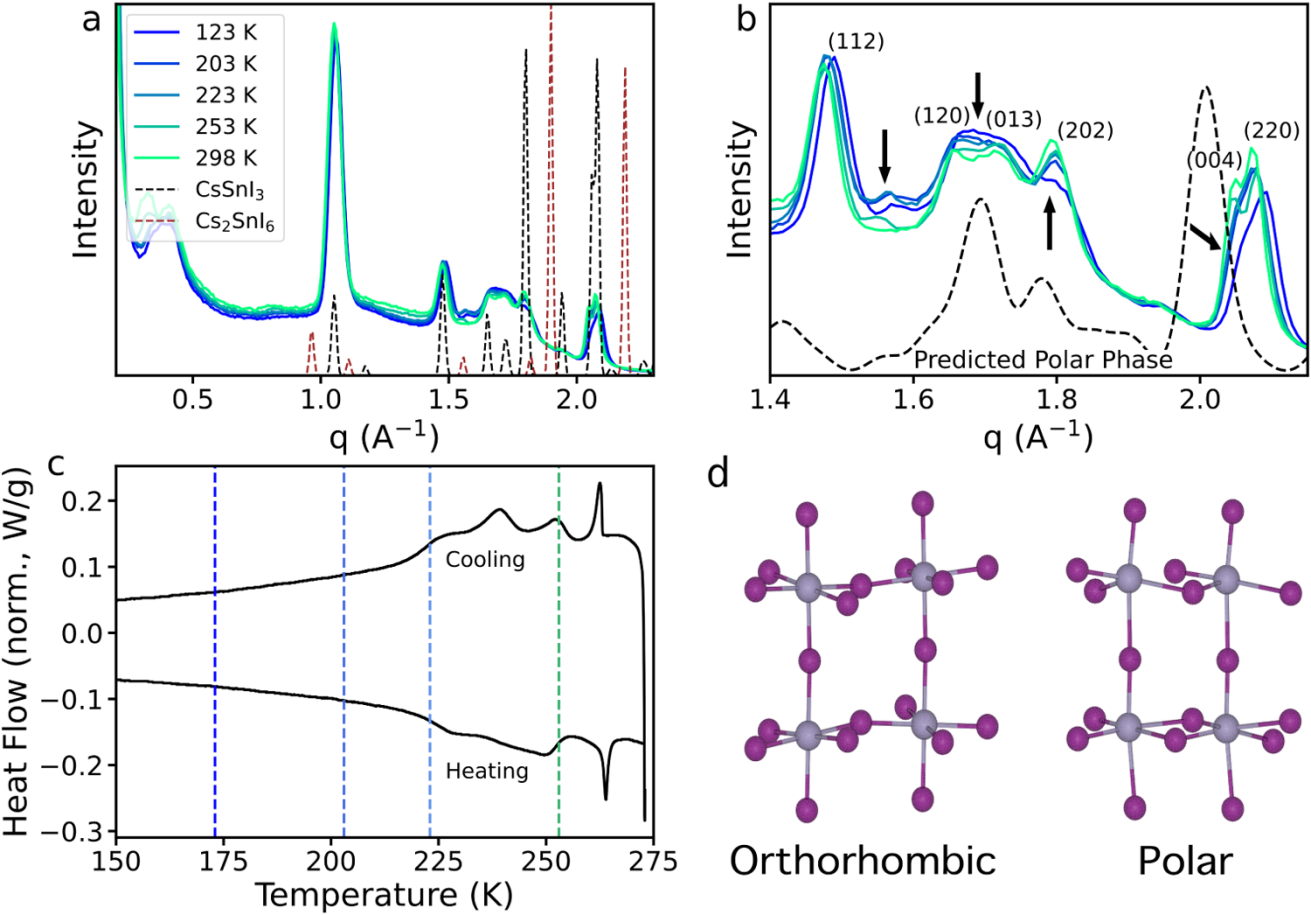
Temperature-dependent XRD measurements of CsSnI_3_ NC film. Panel (a) shows the full collected pattern alongside reference patterns calculated from the bulk structure for CsSnI_3_ and Cs_2_SnI_6_. Panel (b) highlights the changes to the pattern that were observed at low temperatures and shows the calculated pattern from a predicted low-temperature polar phase.^6^ (c) Heat flow for a heating and cooling cycle between 150 and 273 K, where the positive and negative peaks are characteristic for exo- and endothermic events, respectively. Dashed lines denote the temperatures at which XRD measurements were taken. (d) Structural models of the orthorhombic and polar phases. Note the loss of symmetry around the octahedral Sn sites in the polar phase.

To further investigate the source of the unusual PL behavior, we performed temperature-dependent transient absorption (TA) measurements. Figure 4 shows TA data of CsSnI_3_ NCs collected at 295 K (a–c) and 200 K (d–f) using a 400 nm (3.1 eV) pump. Two strong bleaches, each with an adjacent photoinduced absorption (PIA) feature, are present at both temperatures. We assign the lower-energy bleach to the exciton, and HE bleach to the second absorption feature occurring around 600 nm, which also correlates to the HE emission seen below 240 K. These absorption features can be further assigned as transitions between the *J*_h_ = 1/2 valence band to the Sn-derived *J*_e_ = 1/2 split-off conduction band that make up the lower energy exciton transition, and the same *J*_h_ = 1/2 valence band to the *J*_e_ = 3/2 conduction band for the HE feature, based on previous calculations for the electron structure of both tin^33^ and lead^34,35^ halide perovskites. These features can be seen in both the full spectral data (a, d) as well as the time slices (b, e), with the features being slightly sharper in the 200 K data. The bleaches and their adjacent PIA are both red-shifted at 200 K due to the red shift of the features in the steady-state absorbance (see Figure 2). The lifetimes of both PIA features closely track those of their accompanying bleaches, indicating that they both arise from the excited state. At 295 K, the exciton bleach and HE bleach exhibit similar lifetimes, consistent with rapid decay of electrons from the *J*_e_ = 3/2 conduction band state associated with the HE feature to the *J*_e_ = 1/2 conduction band state associated with the exciton transition, with the remaining bleach lifetime driven primarily by holes in the *J*_h_ = 1/2 valence band state common to both transitions. Both bleaches exhibit biexponential decay and were well-fit using eq 2 in the SI (see Table S1 for fit results). At 200 K, we observe a decrease in lifetimes for both bleaches, with the initial decay of the exciton bleach becoming faster than that of the HE bleach (Figures 4f, 6, and S14c). This shows a relative increase in the HE state lifetime at low temperature. Full TA kinetic traces for each temperature out to 7 ns are shown in Figure S13. Additionally, the exciton lifetime does not completely decay to zero (Figure S13), exhibiting an apparent long-lived state which may be gradually decaying above the nanosecond scale. However, the signal in this region is very close to the noise level for our instrument (0.3 mOD), so we cannot rule out the possibility that this residual signal may simply be noise. This is further supported by the adjacent PIA feature exhibiting the same lifetimes, while still reaching zero. The kinetics in Figure 4f have therefore been offset such that both bleaches share the same zero, to allow for easier visual comparison of the kinetic behavior at earlier times.

**Figure 4:**
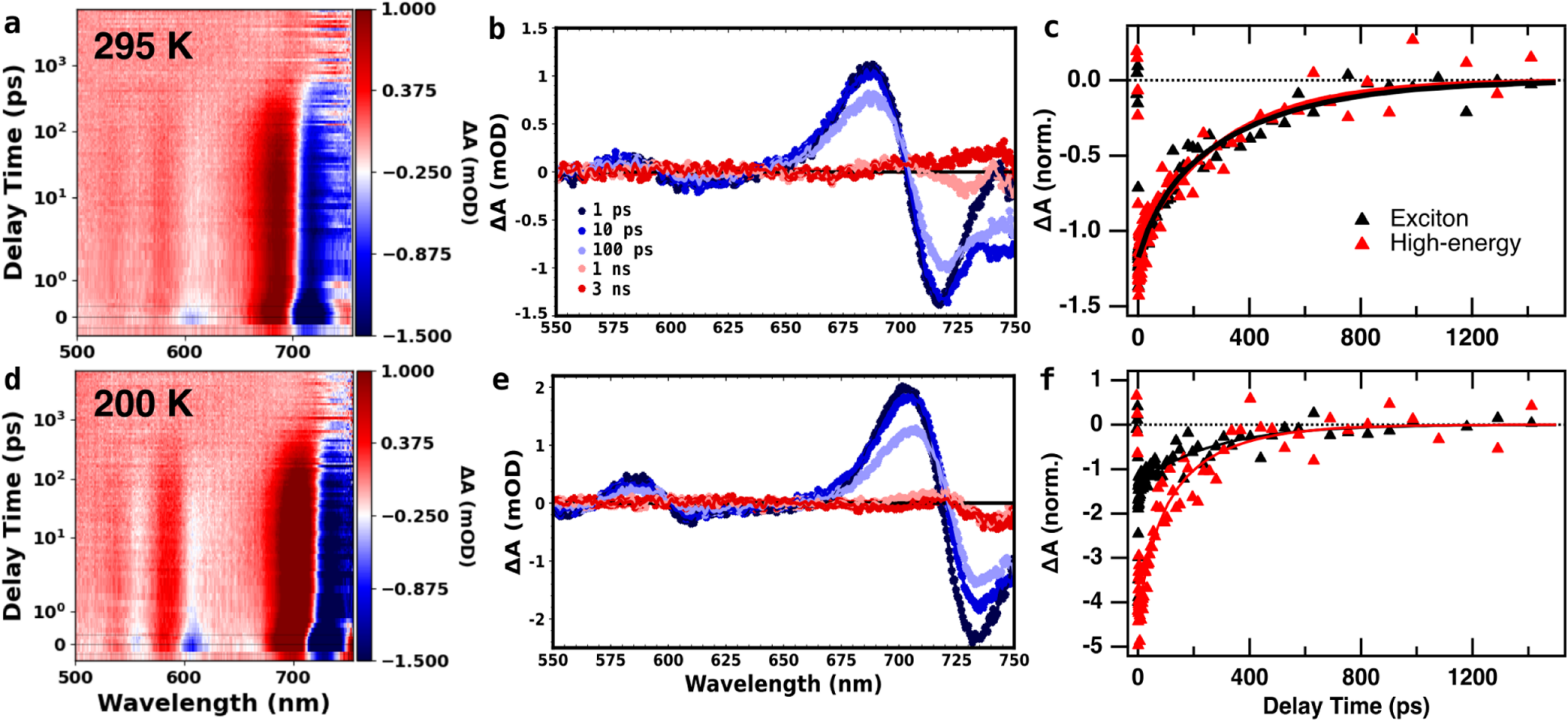
Transient absorption spectroscopy of CsSnI_3_ nanocrystals with a 400 nm (3.1 eV) pump, at 295 K (a–c) and 200 K (d–f). Panels (a and d) show TA spectra, (b and e) show spectral slices at 1 ps, 10 ps, 100 ps, 1 ns, and 3 ns. Panels (c and f) show kinetics traces (normalized at 10 ps) of the exciton bleach (black) and high-energy bleach (red) features from 0 to 1500 ps. Solid lines are biexponential fits to the kinetics data.

Figure 5 reports the TA data for CsSnI_3_ NCs at 200 K excited with a 650 nm (1.9 eV) pump. In this case, the pump wavelength is not short enough to excite the higher energy state. The spectra (a) show similar features to those pumped at 400 nm, with PIA features that are stronger relative to the bleaches, as seen in the time slices (b). However, with this pump wavelength, the kinetics of the two bleaches are essentially identical, with very similar monoexponential decay behavior (c) (full kinetics fit results shown in Table S2, fit using eq 3). These traces represent simple decay behavior of the exciton (radiative decay of electrons from the *J*_e_ = 1/2 conduction band state to the valence band) with bleaching of the HE state resulting from holes in the valence band. This is in contrast to the 400 nm pump data, where the kinetics exhibit biexponential decays due to the additional decay of electrons from the *J*_e_ = 3/2 conduction band state associated with the HE transition to the *J*_e_ = 1/2 conduction band state associated with the exciton transition.

**Figure 5:**
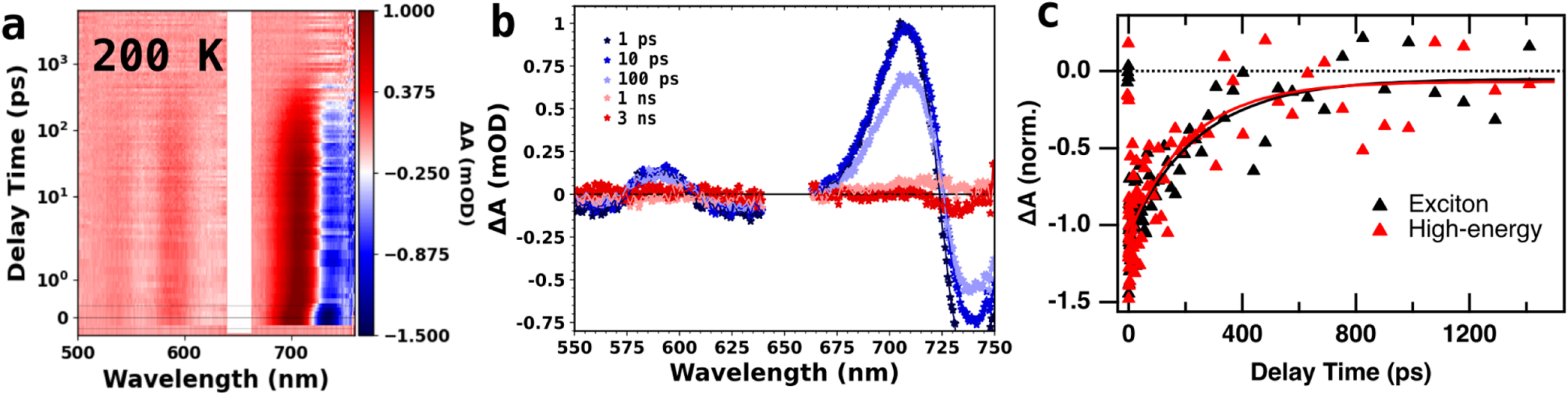
Transient absorption spectroscopy of CsSnI_3_ nanocrystals at 200 K with a 650 nm (1.9 eV) pump. (a) TA spectra. (b) Spectral slices at 1 ps, 10 ps, 100 ps, 1 ns, and 3 ns, with pump scatter removed. (c) Normalized kinetics traces of the exciton bleach (black) and high-energy bleach (red) features from 0 to 1500 ps. Solid lines are monoexponential fits to the kinetics data.

Optical features in TA data can sometimes be convoluted with thermal difference effects resulting from the heating of the sample by the laser. Indeed, several of the features in our TA spectra appear remarkably similar to the thermal difference spectra calculated from our temperature-dependent steady-state absorption data (Figure S16). In order to determine whether thermal effects were influencing our data, we compared TA measurements under identical conditions on two substrates with different thermal conductivities: quartz (3 W/(m K)) and sapphire (46 W/(m K)). Figure S17 shows the normalized exciton bleach kinetics measured on each substrate, with no significant difference observed between them, particularly on the nanosecond time scales where thermal effects would be expected.^36,37^ We therefore conclude there are little to no significant thermal effects in our TA data and thus attribute the decay dynamics to the excited carrier dynamics.

TA spectroscopy is a sum of the contributions from both electron/conduction band and hole/valence band dynamics; i.e., Δ*A* = *f*_e_(*t*)­σ_e_ + *f*_h_(*t*)­σ_h_, where *f*_e_(*t*) and *f*_h_(*t*) are the electron and hole occupation as a function of time and σ_e/h_ are the contributions to the absorption cross section from electrons/holes. As discussed above, the exciton and HE transitions share the same valence band; therefore, we need to deconvolve the valence band dynamics from the HE bleach kinetics in order to isolate the HE electron dynamics. To do this, we normalized the TA kinetics for the HE and exciton at longer delays where any HE electrons have decayed and *f*_e_^HE^(*t* > τ_HE_) = 0, where τ_HE_ is a characteristic time for the HE electrons to relax to the band edge. In this case, the dynamics from the HE and exciton are identical and the difference Δ­(Δ*A*) contains only the dynamics for the HE electron, i.e., we can isolate *f*_e_^HE^(*t*). Figure 6a shows Δ­(Δ*A*)­(*t*) with a 400 nm (3.1 eV) pump at 295 K (black circles) and 200 K (red circles and line). At 295 K, there is no discernible difference between these two features except at very early times, indicating that the TA kinetics of the higher energy state is only sensitive to the hole dynamics at the valence band edge. We attribute this result to subpicosecond cooling of the initially excited HE electron to the band edge, as shown in Figure 6b. In contrast, at 200 K, there is a substantial difference between the exciton bleach and HE bleach at short times (Figure 6a), suggesting that cooling of carriers initially excited to the HE state is slowed in the low-temperature polar phase. Fitting these different spectra with a single exponential yields a lifetime of 80 ps. Additionally, a 650 nm (1.9 eV) pump at 200 K produces no difference between these two bleaches (Figure 6a, blue triangles), indicating that the difference is solely the result of pumping into the HE state and not the result of hole lifetimes. These results, and those of Figures 4 and 5, are consistent with the energy level structure shown in Figure 6b,c, which share a common valence band maximum. We note that the energy spacing between the exciton and the HE transition, Δ ∼ 340 meV, is close to the calculated spacing between the Sn-derived *p*_3/2_ and *p*_1/2_ conduction band levels in bulk cubic phase CsSnI_3_ reported by Huang and Lambrecht^33^ and is consistent with the band structure for the polar phase CsSnI_3_ shown in ref 6. This separation, known as the split-off band parameter, is the likely origin of the splitting between the exciton and HE transition.^34,35^ As such, it is likely that the HE feature is due to the transition from the valence band maximum to the higher energy heavy-electron/light-electron complex characterized by total angular momentum *J* = 3/2, in contrast to the exciton transition, which connects the valence band maximum to the minimum of the conduction bands with total angular momentum *J* = 1/2. We therefore assign the HE state to *J*_e_ = 3/2, and the exciton to *J*_e_ = 1/2. This is similar to the reported assignment of a similarly spaced high-energy feature observed in TA spectra for FASnI_3_ nanocrystals.^12^

**Figure 6:**
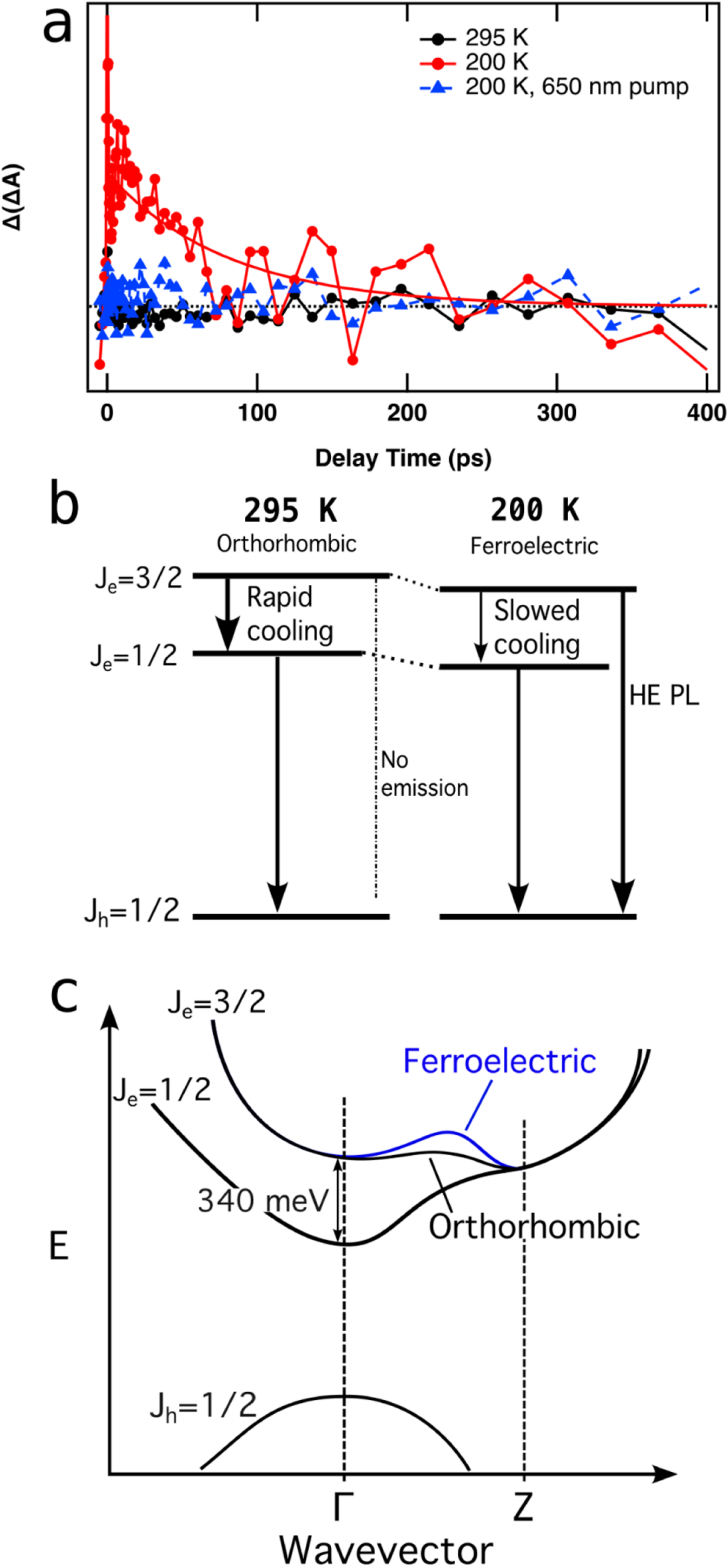
(a) Difference between exciton and high-energy bleaches normalized at long lifetimes, with exponential fit to the data. (b) Scheme depicting changes in kinetics behavior of bands between 295 and 200 K. (c) Simple scheme depicting hypothesized increased barrier in the band structure in the ferroelectric phase vs the orthorhombic phase. This barrier would lead to increased carrier lifetimes in the Γ valley, which is consistent with our TA data. State labels in panels (b and c) are from electronic structure calculations.^33–35^

We attribute the increased lifetime shown here to slowed carrier cooling to the exciton state as a result of the phase transition from orthorhombic to a lower-symmetry, possibly polar, phase. We hypothesize that in the low-temperature phase, the energy band pathway connecting the upper conduction band minimum and the lowest conduction band minimum in k-space traverses a region of higher energy states, which could present a barrier to carrier relaxation. Such a situation was reported in GaAs,^38^ where excitation into the upper conduction X or L valleys results in reduced carrier relaxation within the Γ valley. As such, the band structure of the low-temperature phase of CsSnI_3_ schematically drawn in Figure 6c could result in a reduced cooling rate from the HE state to the exciton state at k = 0, allowing for radiative emission from the HE state to be competitive.

## Discussion

Numerous possible sources of dual emission or hot carrier emissions have been reported in the literature. We now consider each possibility in the context of the CsSnI_3_ NC system reported here. Previously reported sources of dual emission include: defect states, emissive dopants, mixed compositions and/or morphologies, and mixed phase materials. Defects are known to produce dual emission in semiconductor systems, usually from a defect state below the bandgap, where charge carriers become trapped.^39–42^ The emission we observe, in contrast, is both above bandgap and tied to existing states in the NCs, and we do not observe any new states in our TA data below 240 K. Halide perovskites are additionally known to be highly defect tolerant;^1,2,43–48^ thus we conclude defects are not the likely source of the hot-emission we observe. Dopants are another possible source of dual emission, particularly in NC systems.^49–53^ Due to the high-purity reagents used (see Experimental) and lack of evidence for dopant states in our PL and TA data, we are confident that no meaningful unintentional dopants are present. Mixed composition or varied morphology is also known to yield dual emission from semiconductor NCs, exemplified by the CdX tetrapods reported by the Alivisatos group.^54–57^ However, XRD and TEM show no evidence of additional phases or morphologies in our sample, strongly indicating that our nanocrystals are single-composition and single-morphology. Our optical data are also consistent with a single-phase material, exhibiting simple dynamic behavior until the phase transition temperature. 2D nanosheets of [R–NH_3_]_2_SnI_4_ are reported to form under similar conditions to those used to synthesize CsSnI_3_ NCs,^18,58,59^ and may be present as a minority species in our samples. In order to investigate this, we synthesized [R–NH_3_]_2_SnI_4_ 2D nanosheets following the procedure of Gahlot et al.,^58^ and mixed these at various ratios. We observed the nanosheets to be colloidally unstable and rapidly crash out of solution in an attempt to make optical measurements. Figure S20 shows the low-temperature photoluminescence of a 50/50 mixture of nanosheets and CsSnI_3_ NCs measured at 120 K. If trace 2D nanosheets were the source of our high-energy PL, we would expect to see the same peak with higher intensity for this mixture. Instead, two slightly different peaks and different behaviors of peak intensity ratios can be clearly seen for the mixture and the CsSnI_3_ NCs, indicating that the nanosheets emit at a different wavelength at low temperatures. We therefore conclude that these are not the source of the emission we observe in our CsSnI_3_ NCs samples, and it is highly unlikely that any of these 2D nanosheets are present.

Different crystal phases of cesium tin iodide are known to exhibit different emission behaviors. Notably, NCs of Cs_2_SnI_6_ exhibit PL around 1.5 eV (800 nm),^60,61^ at much lower energy than that observed here. CsI or SnI_2_ are additional possible contaminants or secondary phases with higher lying optical transitions, but neither of them absorbs light in the 500–700 nm region relevant to the novel behavior observed here.^62,63^ Additionally, our XRD data show no evidence of any of these compositions nor the rarer Cs_4_SnI_6_ phase at room temperature or low temperature (see Figures 3 and S12), leading us to dismiss this possibility as well. An amorphous phase is also unlikely; our XRD data have a low background at room temperature, and it further decreases upon cooling. Previous work has attributed dual emission in halide perovskites to the presence of molecularly disordered domains at low temperature.^64^ Our TA data indicate this is likely not the case here as the same states exist at room temperature and low temperature, with a slight red shift upon cooling (Figures 4 and 5). Additionally, the similar red-shift behavior observed for both states upon cooling (Figure 2) strongly suggests they emerge from the same crystal phase. Further work employing pair distribution function measurements could conclusively determine the presence or absence of such disordered domains at low temperature.

Our results additionally contrast with previously observed hot carrier behavior.^10–13,65,66^ Previously, observations of slowed cooling are on the scale of 10 ps, in contrast to the 80 ps lifetime we observe here. Furthermore, with the exception of ref 12, which observed a double peak structure (similar to the ones we report) in the TA spectra of FASnI_3_ NCs, prior reports observed hot carrier PL as a high-energy tail on the band-edge emission, which emerges under high pump fluences. The dual peaks in our PL data and the fact that we observe this behavior at low fluences suggest that a different phenomenon governs the observed behavior here. We therefore dismiss the traditional hot-phonon bottleneck within the *J*_e_ = 1/2 conduction bands as a major cause.^67^ This leads to our conclusion that the phase transition is ultimately responsible for the new behavior.

There are two possible causes of the slowed carrier cooling we observe: either the radiative recombination rate from the HE state increases or the cooling rate to the band edge decreases. Since in the TA measurements we find that cooling is slowed in the low-temperature phase, we conclude that the latter is occurring. This correlates with the changes we observe in the crystal structure at a low temperature. Changing the structure can lead to changes to vibrational modes, including density of modes, frequencies of modes, and population of modes (this in particular will decrease as the temperature decreases). We propose three possible hypotheses for how this change in structure leads to the changes in relaxation dynamics that we observe: (1) As mentioned in the Results section, the band structure changes in the ferroelectric phase (Figure 6c), which creates a barrier for the relaxation of carriers from the *J*_e_ = 3/2 conduction band state to the *J*_e_ = 1/2 conduction band state associated with the exciton. In fact, we find that the intensity of the HE PL increases with decreasing temperatures and the increase in PL can be modeled assuming an activation barrier to quench the PL. We estimate the activation barrier is roughly 76 meV based on an Arrhenius fit using eq 1 (Figure S11) to the temperature-dependent integrated PL intensity. We estimated the barrier in the band structure sketched in Figure 6b for ferroelectric CsSnI_3_ using computations by Swift and Lyons,^6^ and find reasonable agreement. Thus, such a barrier could slow the rate of carrier relaxation sufficiently to allow competition from the radiative relaxation to the ground state. (2) In ref 6 it was predicted that this phase transition leads to a pronounced Rashba effect, depicted schematically in Figure S21. The Rashba splitting may occur within both the *J*_e_ = 1/2 and *J*_e_ = 3/2 conduction band groupings, leading to offset conduction band minima. (3) It is also possible that in the ferroelectric phase, polaron formation may become more pronounced,^68,69^ leading to reduced carrier scattering and longer carrier lifetimes for the excited-state carriers.

## Conclusions

We have observed novel low-temperature properties from a homogeneous, phase-pure sample of CsSnI_3_ nanocrystals. Upon cooling, these nanocrystals undergo a phase transition, possibly to the monoclinic polar phase predicted by Swift and Lyons.^6^ Their steady-state absorbance and PL both gradually red-shift upon cooling; with a change in the trend around 240 K we correlate to the phase transition. Most interesting, a new, high-energy PL peak emerges below 240 K concurrently with the exciton PL. This peak is directly adjacent to the high-energy feature in the absorption and appears to be photoluminescence from this state, which we assign as the transition from valence band maximum to the higher energy heavy-electron/light-electron complex characterized by *J*_e_ = 3/2.^12,33^ Our transient absorption data support this conclusion, revealing an increase in the excited state lifetime at low temperature and showing that the same states are present at room temperature. These results provide insight into the potential of novel halide perovskite-related phases for hot carrier utilization as well as the possible role of the phase on carrier dynamics. Further research is needed to determine the exact nature of this low-temperature phase and target methods for raising the transition temperature closer to room temperature, as well as understanding the impacts on excited state lifetimes and carrier dynamics.

## Experimental Section

### Materials

Cesium carbonate (99.9%), oleic acid (OA, 90%), oleylamine (OLA, 70%), 1-octadecene (ODE, 90%), toluene (anhydrous, 99.8%), hexane (anhydrous, 95%), and octane (anhydrous, ≥99%) were purchased from Sigma-Aldrich. OA, OLA, and ODE were dried under a vacuum at 120 °C for 4 h and stored under nitrogen in a glovebox prior to use. SnI_2_ (ultradry, 99.999%) was purchased from Alfa Aesar and used as-is under an inert atmosphere only.

### Synthesis of CsSnI_3_ Nanocrystals

CsSnI_3_ NCs were synthesized following the hot injection procedure reported by Gahlot et al.^18^ The cesium oleate (0.222 M) solution used for hot injection was freshly prepared for each synthesis. All glassware was oven-dried. 326 mg (1 mmol) cesium carbonate, 1 mL (3.2 mmol) oleic acid, and 8 mL octadecene were stirred under vacuum for 1 h at 120 °C and then heated to 150 °C under nitrogen to complete dissolution. The resulting clear, pale yellow solution was used for the injection.

In a nitrogen glovebox, 0.75 g (2 mmol) of SnI_2_, 0.63 mL (2 mmol) of oleic acid, 0.66 mL (2 mmol) of oleylamine, and 5 mL of octadecene were sealed in a round-bottom flask. This was transferred to a Schlenk line and degassed for 10 min at room temperature and then 35 min at 105 °C to dissolve all SnI_2_. The solution was then heated to 200 °C under nitrogen and 2.8 mL (0.622 mmol) 0.222 M hot cesium oleate solution (completely liquid and transparent) was swiftly injected, then immediately quenched in an ice–water bath. Faster quench times were found to reduce the ‘tail’ in the absorbance spectrum. Solid or congealed cesium oleate during injection led to impurities. In a glovebox, the crude solution was centrifuged at 7000 rcf for 3 min. The precipitate was resuspended in 4 mL of toluene, then centrifuged at 18,500 rcf for 5 min. The resulting black/brown precipitate was again resuspended in 5 mL of toluene and stored under a nitrogen atmosphere before using for further measurements.

For steady-state absorbance and PL measurements, samples were diluted in toluene to 0.2–0.6 optical density, usually requiring a factor of 20 dilution.

### Characterization

Absorbance spectra were measured using a Cary 6000 UV–vis spectrometer with dilute sample solutions in toluene or 8:1 hexanes:octane in 2 mm cuvettes. Photoluminescence data were collected by using a Princeton Instruments spectrometer and 10 mm PL cuvettes. For both Abs and PL, low-temperature measurements were performed using the same instrumentation in conjunction with an Oxford Instruments Optistat DN cryostat, using liquid nitrogen to cool the sample. Due to the low temperatures involved, spin-coated sample films were used instead of solutions. Spin-coating 20 uL of a 1/5 diluted solution of 8:1 hexanes:octane at 2000 rpm for 20 s yielded the best film optical quality. Additional samples were prepared in a polystyrene matrix by adding 200 uL of neat NC solution to 5 wt % polystyrene in toluene and then spin-coated using identical conditions. During the measurements, samples were allowed to stabilize at the measured temperature for 15 min before collecting data.

Benchtop XRD measurements were made using a Bruker D8 Discover system, with samples sealed between two layers of Kapton tape. TEM samples were prepared by drop-casting a 1/10 dilution of NCs in toluene onto ultrathin carbon type A grids (Ted Pella) under an inert atmosphere. Grids were then dried under vacuum for 18–20 h and plasma cleaned for 20 s at 40 W using a nitrogen/hydrogen plasma (ibss Group GV10x Downstream Asher) prior to imaging. TEM, STEM, and HR-STEM images were acquired using a Thermo Scientific Spectra 200 G2 probe-corrected S/TEM, Cs correction. Differential scanning calorimetry (DSC) measurements were performed on a calibrated Discovery 25 TA Instruments with a cryogenic cooling system mounted to allow it to reach temperatures down to 100 K (−160 °C). NC films were deposited and enclosed in hermetically sealed Tzero aluminum pans prepared in an Ar glovebox, with a reference pan prepared in an Ar atmosphere. The samples were heated and cooled at 2 K/min between the temperatures of 100 and 300 K for an initial survey and between 150 and 300 at 10 K/min for all other samples.

#### Transient Absorption Measurements

A Coherent Astrella Ti:sapphire regenerative amplifier with a repetition rate of 1 kHz and a fundamental wavelength of 800 nm (approximately 100 fs pulse width) was used for ultrafast transient absorption experiments. The 650 nm (6.3 μW, 55 nJ/mm^2^) pump pulses were generated in an optical parametric amplifier (Quantronix PalitraDuo), and the 400 nm pump pulses were generated by doubling the fundamental. The probe pulse (λ probe = 420 to 760 nm) was generated by focusing approximately 10 μJ of the Astrella output into a 2.5 mm sapphire plate. The probe pulse was focused at the sample, spatially overlapped with the pump, and a mechanical delay stage was used to delay the probe pulse relative to the pump. The time window for the experiment is 7 ns. Several artifacts were observed when using simple NC films (no polystyrene) or NC solutions, so thin films of NCs in a polystyrene matrix were used for all measurements to prevent charging and other particle–particle interactions. Power dependence of lifetimes was measured to ensure that all measurements were performed in the linear regime (Figures S18 and S19). Data were background-subtracted and chirp-corrected prior to analysis using in-house code. The KiMoPack python package was used for data processing and analysis.^70^

#### Wide-Angle X-ray Scattering (WAXS) Measurements

Variable temperature transmission WAXS measurements were performed on a Xenocs Xeuss 3.0 SAXS/WAXS instrument with a Cu Kα source (λ = 1.54 Å) and Dectris Eiger2 R 1 M area detector. Samples were drop cast onto a Kapton film and sealed with Kapton tape to prevent oxidation. The tape was mounted on a Linkam HFSX 350 stage in vertical orientation for variable temperature measurements. Measurement of the sample–detector distance and subsequent 2θ calibration was performed using a LaB_6_ standard reference. All data reduction steps, including Q-space conversion, detector masking, and azimuthal integration to obtain a 1-D diffraction pattern, were performed by using the Xenocs XSACT software.

#### heXRD Measurements

X-ray diffraction and X-ray total scattering data were collected at beamline 28-ID-2 at the National Synchrotron Light Source II. Drop-cast NC films on Kapton substrates were measured at room temperature by using monochromatic X-rays at 68 keV (λ = 0.1819 Å).

## Supplementary Material



## Notes

Additional optical characterization, tables of kinetics fits, fitting equations, and supporting TA figures (PDF)
