# Supplementary material for: Dual Photoluminescence in Low-Temperature Phase of CsSnI3 Nanocrystals
Source: J Am Chem Soc. 2025 Aug 6;147(33):30436–46. doi: 10.1021/jacs.5c10595 (PMC12371897; doi:10.1021/jacs.5c10595)
Supplement: Supplementary file 1 [file ja5c10595_si_001.pdf]

Supporting Information for Dual Photoluminescence in Low-temperature Phase of $CsSnI_3$ Nanocrystals

Kyle T. Kluherz,[†] Jacob L. Shelton,[†] Nicholas J. Weadock,[‡] Noemi Leick,[†] Peter C. Sercel,[¶] and Matthew C. Beard[*,†]

†*National Renewable Energy Laboratory, Golden, CO 80401, USA.*

‡*University of Colorado Boulder, Boulder, CO 80303, USA.*

¶*Center for Hybrid Organic Inorganic Semiconductors for Energy, Golden, CO 80401, USA*

E-mail: Matt.Beard@nrel.gov

List of Figures

S1 Extended range absorption and PL 3

S2 Electron diffraction patterns 4

S3 1st & 2nd derivatives of the Abs curve 4

S4 Temperature-dependent absorption over extended temperature range 5

S5 Exciton peak scatterplot 6

S6 Exciton absorption peak across temperatures 6

S7 Comparison of heating & cooling cycles in absorbance data 7

S8 Absorption wavelength and OD as a function of temperature 8

S9 Additional temperature-dependent PL spectra 9

S10 Additional temperature-dependent PL scatterplots 10

S11 Arrhenius fit plot of integrated PL intensity as a function of temperature . . 11
S12 XRD data with reference patterns for $CsSnI_3$ and Cs_4SnI_6 11
S13 Long time plots of bleach kinetics – 400 nm pump 12
S14 Log plots of bleach kinetics – 400 nm pump 13
S15 Log plot of bleach kinetics – 650 nm pump 14
S16 Comparison of TA data with thermal difference spectrum 14
S17 TA substrate comparison 15
S18 TA power dependence scatterplot 16
S19 TA power dependence kinetics 16
S20 2D nanosheets / 3D nanocrystals mixture 17
S21 Rashba splitting band structure diagram 18

List of Tables

S1 Fit results to kinetics traces of exciton & high-energy bleaches for 400 nm pump data. 8
S2 Fit results to kinetics traces of exciton & high-energy bleaches for 650 nm pump data 8

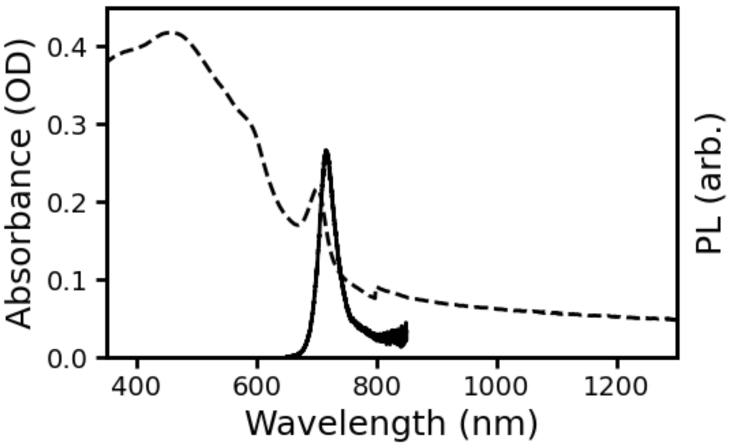

Figure S1: Absorption (dashed) and PL (solid) data of $CsSnI_3$ NCs in a toluene solution at 298 K plotted over an extended wavelength range. The notch at 800 nm is an artifact from the grating changeover on the instrument.

$$I = \frac{A}{1 + Be^{-\epsilon_a/k_bT}} \tag{1}$$

$$\Delta A = y_0 + A_1e^{-x/\tau_1} + A_2e^{-x/\tau_2} \tag{2}$$

$$\Delta A = y_0 + Ae^{-x/\tau} \tag{3}$$

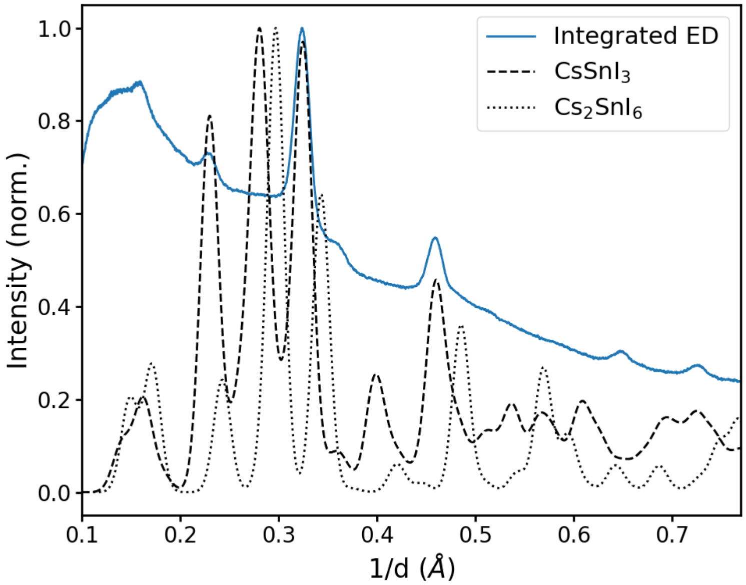

Figure S2: Ring-integrated electron diffraction pattern of $CsSnI_3$ NCs at room temperature, compared with patterns calculated from reference structures (ICSD Collection Codes 69996 ($CsSnI_3$) & 760462 (Cs_2SnI_6)).

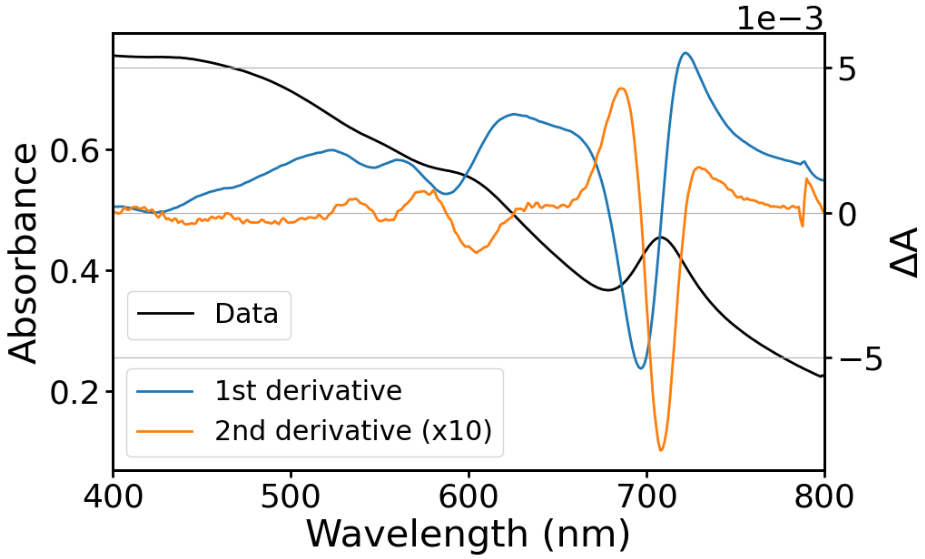

Figure S3: 298 K Steady-state absorbance of $CsSnI_3$ NCs in toluene solution alongside 1st & 2nd derivatives of the Abs curve.

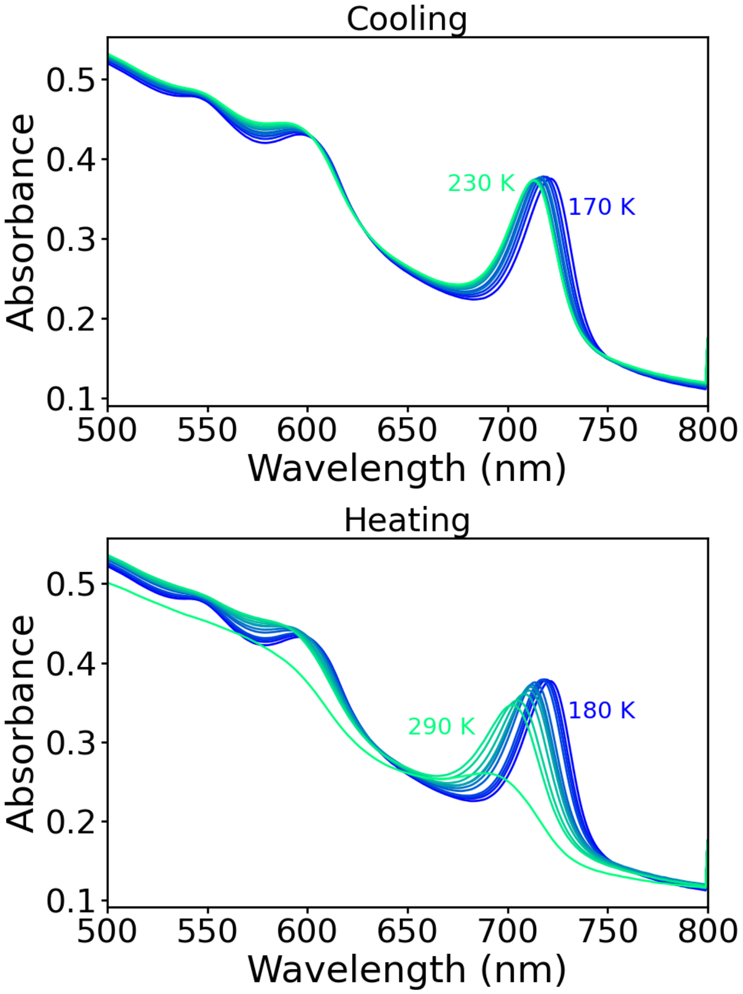

Figure S4: Temperature-dependent absorption measurements over an extended temperature range. Behavior was found to be consistent with the trends observed in a narrower temperature range.

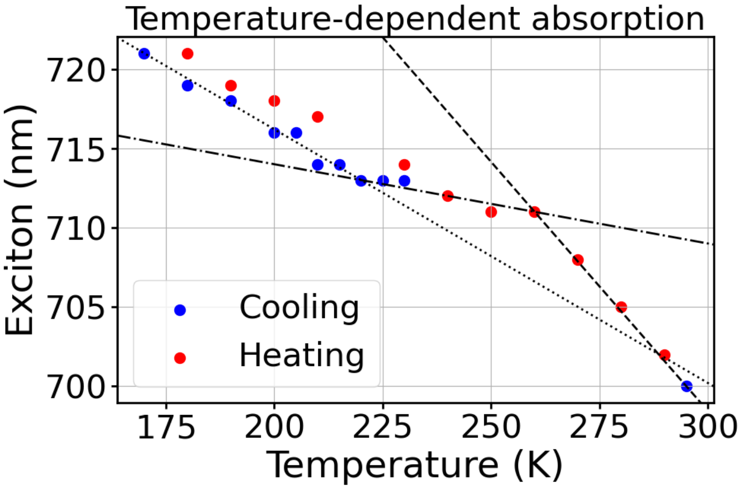

Figure S5: Exciton peak position in absorption data as a function of temperature. Lines of best fit roughly describe 3 regimes of behavior as the sample is cooled.

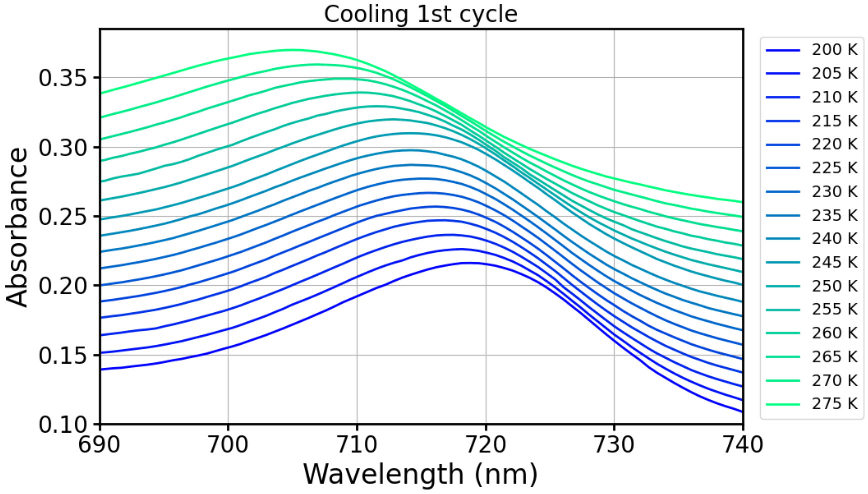

Figure S6: Exciton absorption peak across temperature scan. Note the brief halt in peak shifts around 240 K.

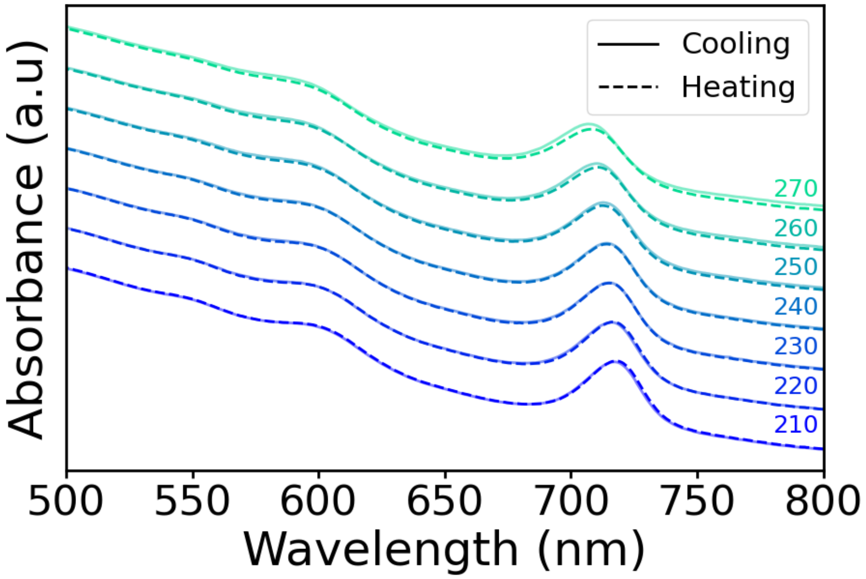

(a)

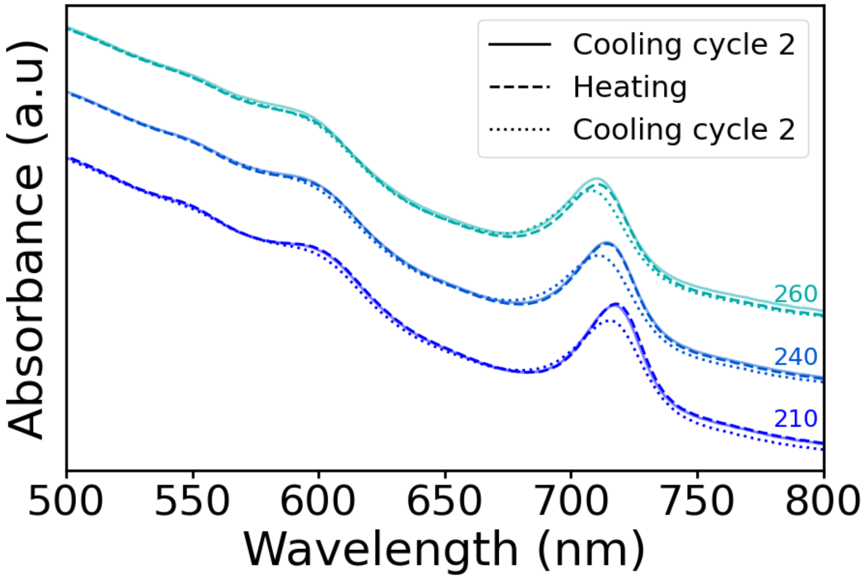

(b)

Figure S7: Comparison of absorbance during cooling and heating (a) and a second cooling cycle (b). Initial cooling and heating yielded highly similar results, with a reduction in exciton peak absorption and variation in position upon second cooling cycle.

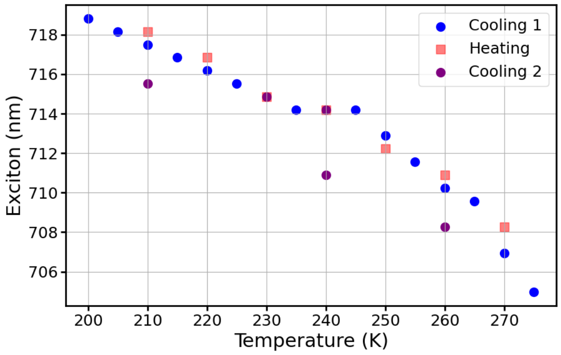

(a)

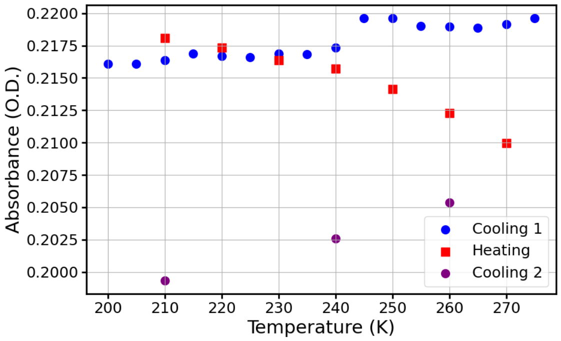

(b)

Figure S8: Exciton absorption peak wavelength (a) and optical density (b) as a function of temperature across cooling-heating-cooling cycles.

Table S1: Fit results to kinetics traces of exciton & high-energy bleaches for 400 nm pump data.

		**295 K**	**240 K**	**200 K**
Exciton	y_0 (mOD)	0	0	-0.16
	A_1 (mOD)	-0.902 ± 0.198	-1.3 ± 1.29	-1.32 ± 0.06
	A_2 (mOD)	-0.277 ± 0.193	-1.4 ± 1.28	-0.44 ± 0.147
	τ_1 (ps)	371 ± 71	341 ± 177	270 ± 37
	τ_2 (ps)	75 ± 52	124 ± 63	3.6 ± 2.3
	χ^2	0.53	1.82	3.11
High-Energy	y_0 (mOD)	0	0	0
	A_1 (mOD)	-0.098 ± 0.007	-0.285 ± 0.004	-0.123 ± 0.136
	A_2 (mOD)	-0.022 ± 0.009		-0.087 ± 0.134
	τ_1 (ps)	308 ± 46	225 ± 12	211 ± 151
	τ_2 (ps)	9 ± 9		62 ± 70
	χ^2	0.02	0.038	0.06

Table S2: Fit results to kinetics traces of exciton & high-energy bleaches for 650 nm pump data. *240 K data were fit using the accompanying PIA absorption feature adjacent to the exciton bleach due to the high noise in the bleach data. High-energy bleaches were not resolvable above the noise in both the 295 K and 240 K data.

		**295 K**	**240 K***	**200 K**
Exciton	y_0 (mOD)	0	0	-0.003
	A (mOD)	-0.447 ± 0.009	-0.5270 ± 0.0028	-0.67 ± 0.027
	τ (ps)	326 ± 27	412 ± 9	248 ± 32
	χ^2	0.27	0.3	0.97
High-Energy	y_0 (mOD)			-0.005
	A (mOD)			-0.067 ± 0.004
	τ (ps)			214 ± 41
	χ^2			0.02

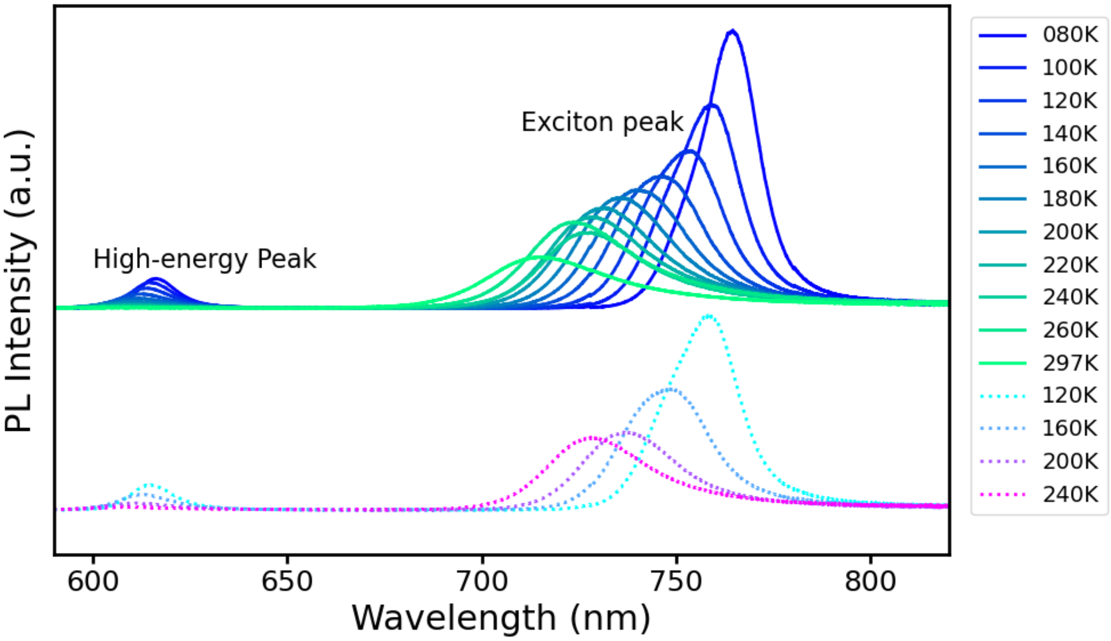

(a)

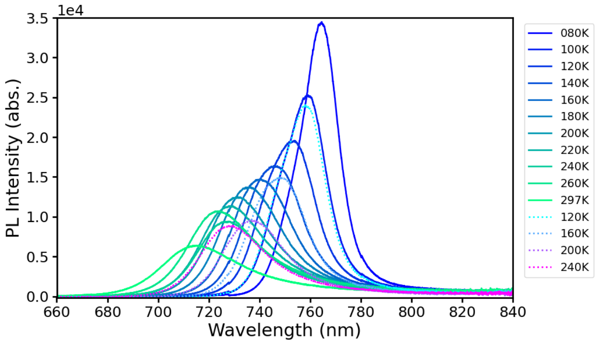

(b) Exciton

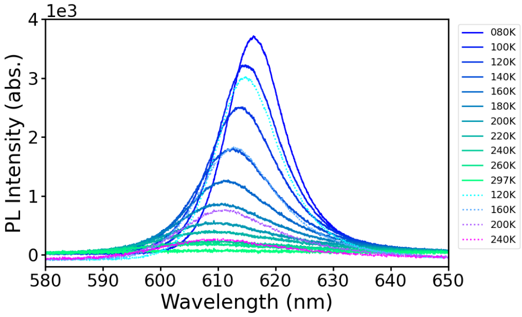

(c) High-energy

Figure S9: Photoluminescence spectra of $CsSnI_3$ NC film as a function of temperature. Solid lines were collected during cooling from room temperature, dotted lines were collected heating from 80 K.

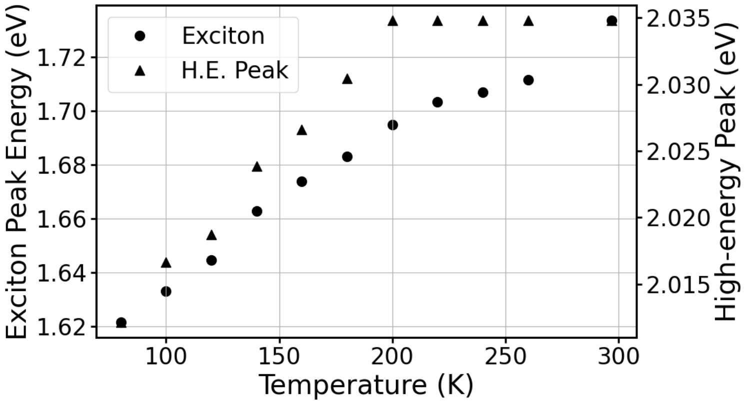

(a)

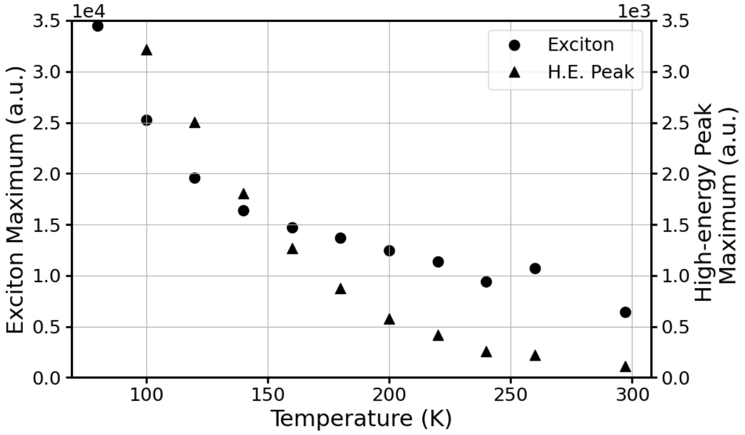

(b)

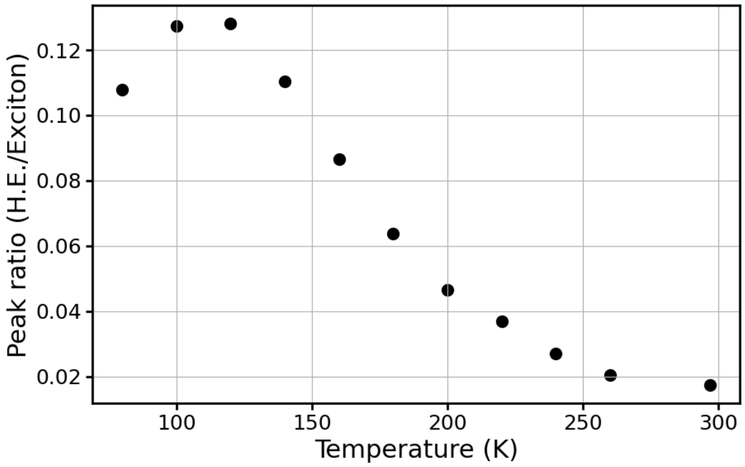

(c)

Figure S10: (a) PL peak energies as a function of temperature. Note that peak 2 only begins to gain sufficient strength below 240 K. (b) PL peak intensities at different temperatures. (c) Ratio of integrated peak intensities as a function of temperature.

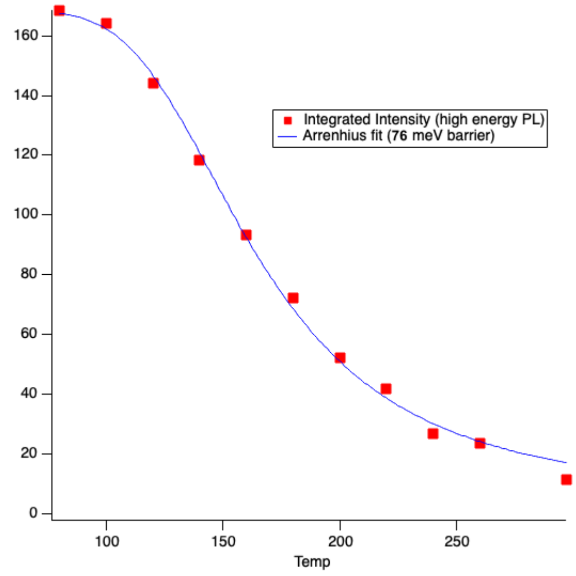

Figure S11: Integrated intensity of high-energy PL emission as a function of temperature, fitted to the Arrhenius equation. This fit yielded a 76±3 meV activation barrier for k-space relaxation.

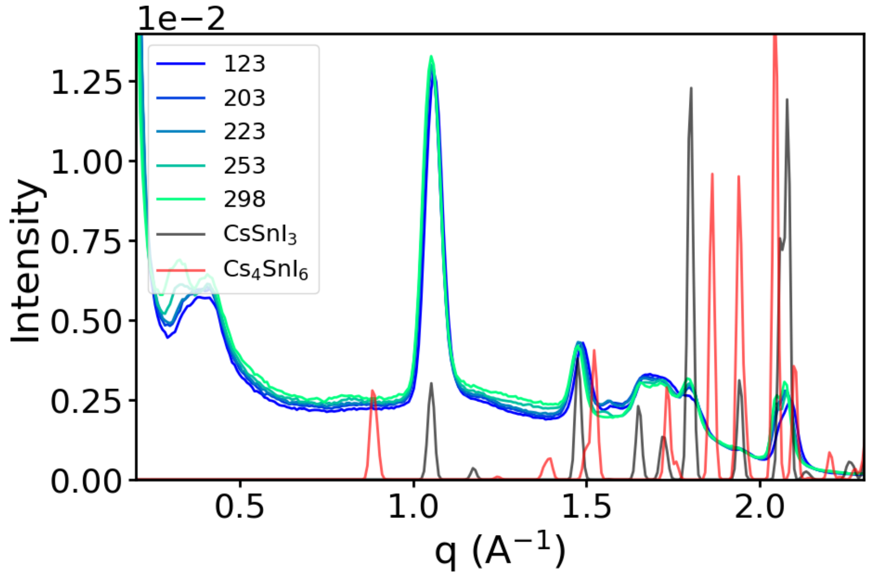

Figure S12: Comparison of low-temperature XRD data with reference patterns for $CsSnI_3$ (ICSD# 69996) and Cs_4SnI_6 (ICSD# 434645) phases.

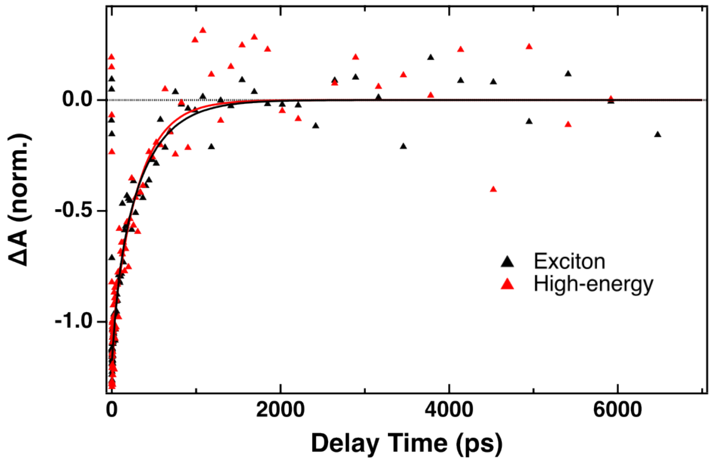

(a) 295 K

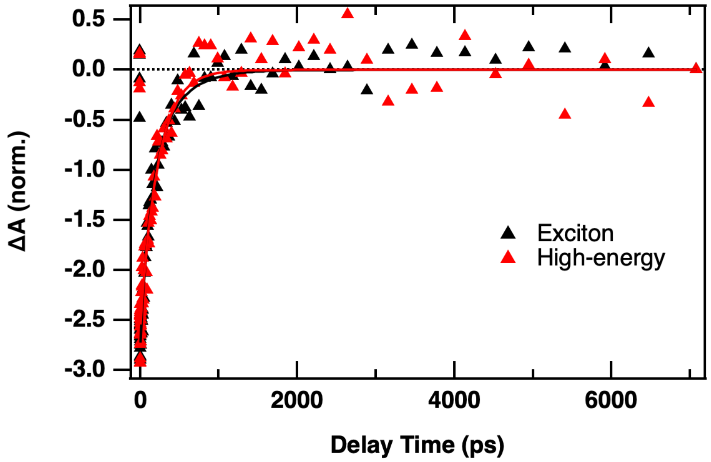

(b) 240 K

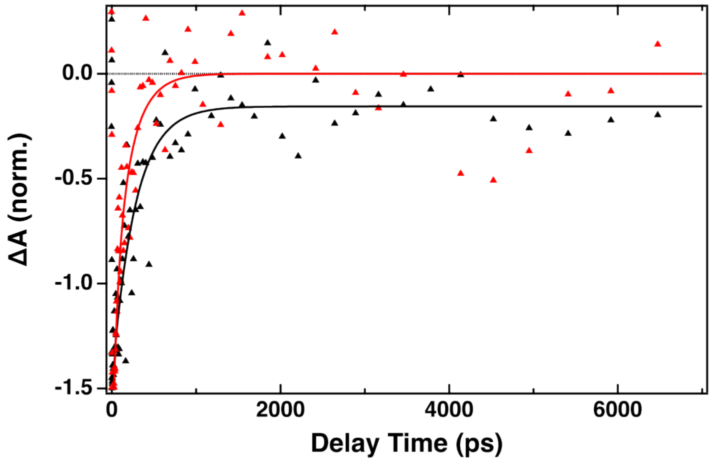

(c) 200 K

Figure S13: Plots of bleach kinetics at 295 K, 240 K, and 200 K out to 7.5 ns of $CsSnI_3$ NCs with a 400 nm pump comparing exciton and high-energy bleaches.

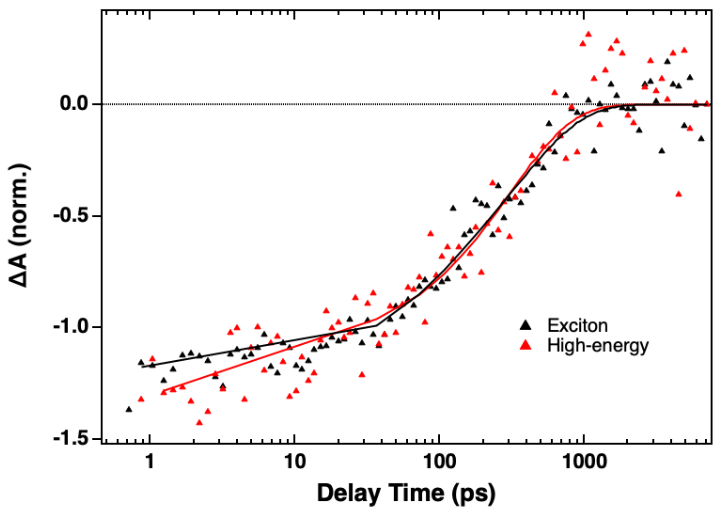

(a) 295 K

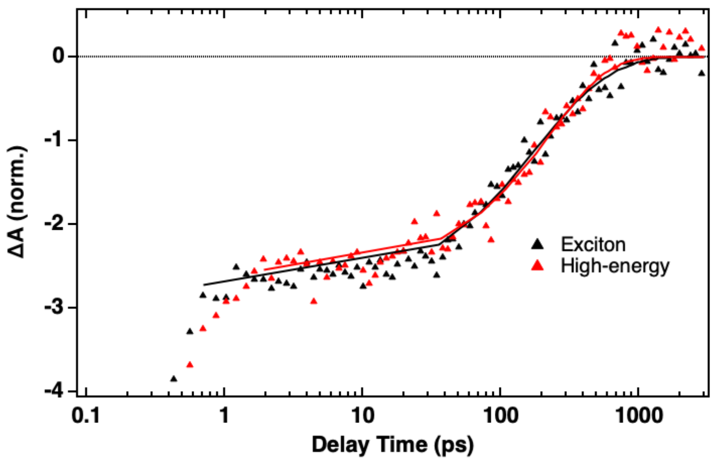

(b) 240 K

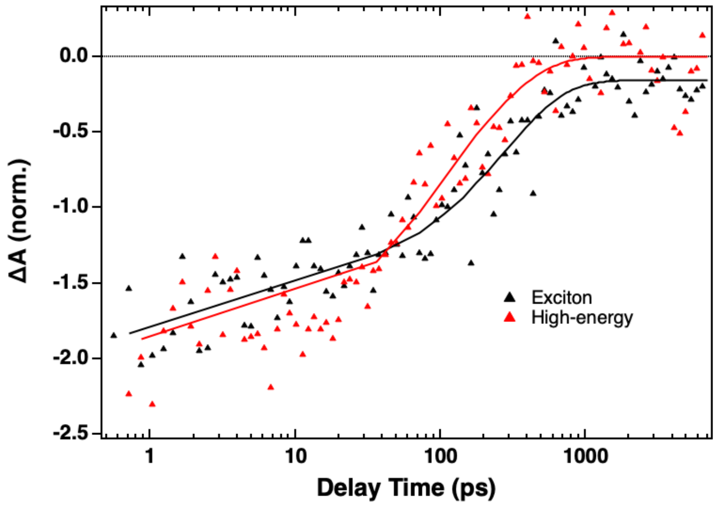

(c) 200 K

Figure S14: Log plots of bleach kinetics at 295 K, 240 K, and 200 K of $CsSnI_3$ NCs with a 400 nm pump comparing exciton and high-energy bleaches.

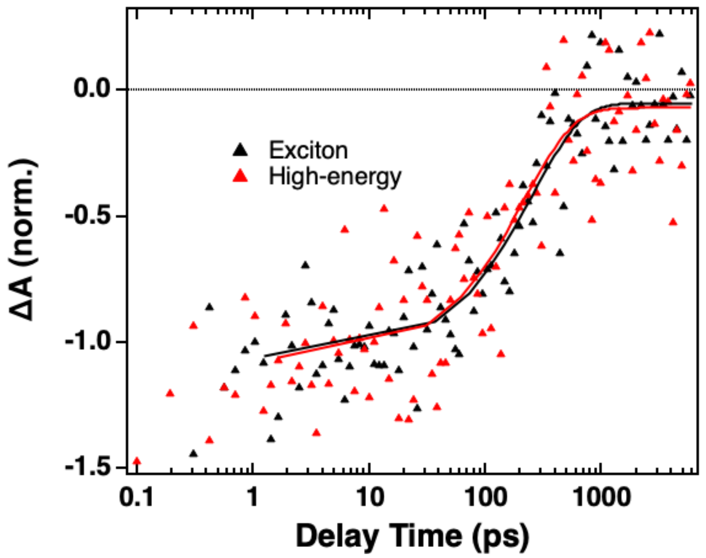

Figure S15: Log plot of bleach kinetics at 200 K of $CsSnI_3$ NCs with a 650 nm pump comparing exciton and high-energy bleaches.

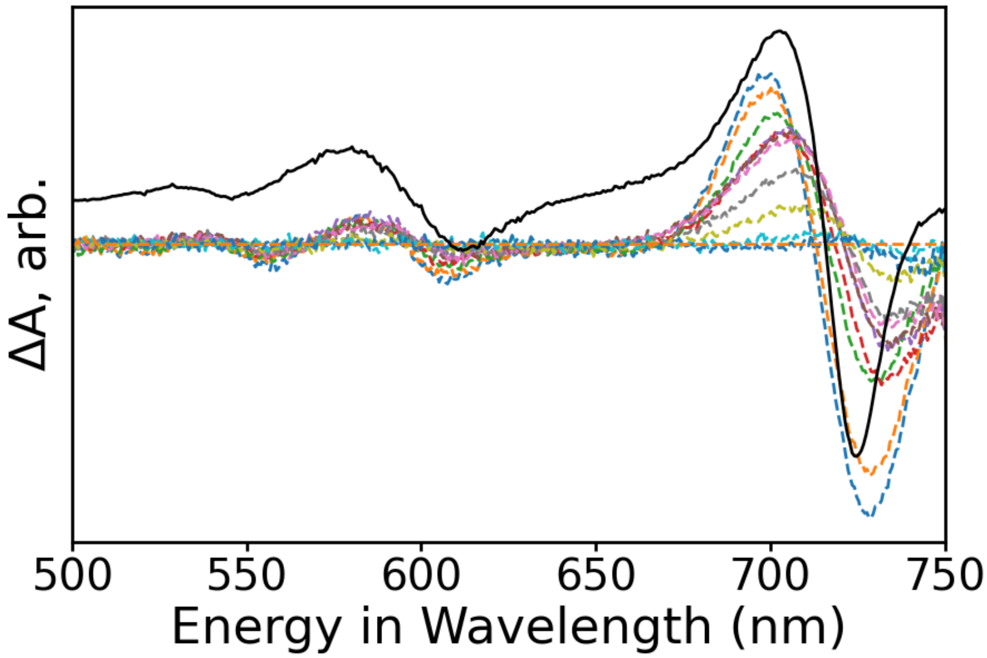

Figure S16: Comparison of 200 K transient absorption data (dashed lines, colored) and thermal difference spectrum (black) calculated from temperature-dependent absorption data (225 K - 200 K). The similarity between the two types of spectra prompted us to investigate the possibility of thermal effects in our TA data.

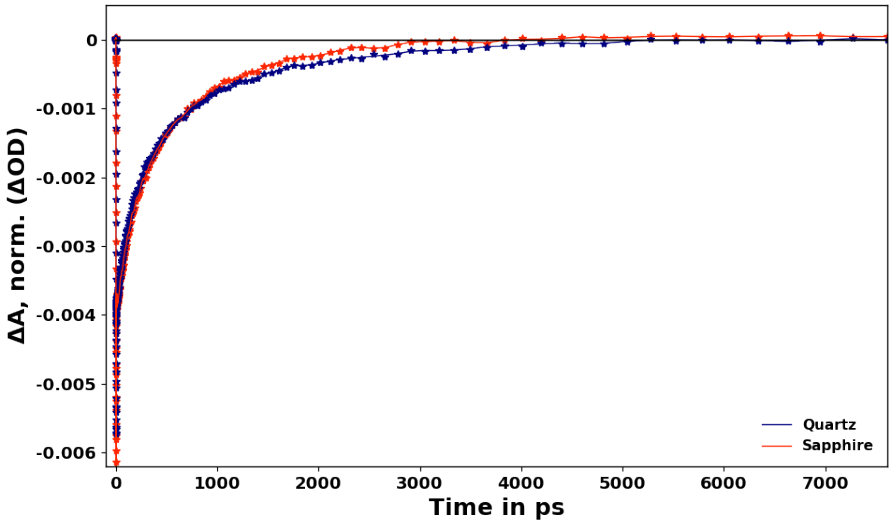

(a)

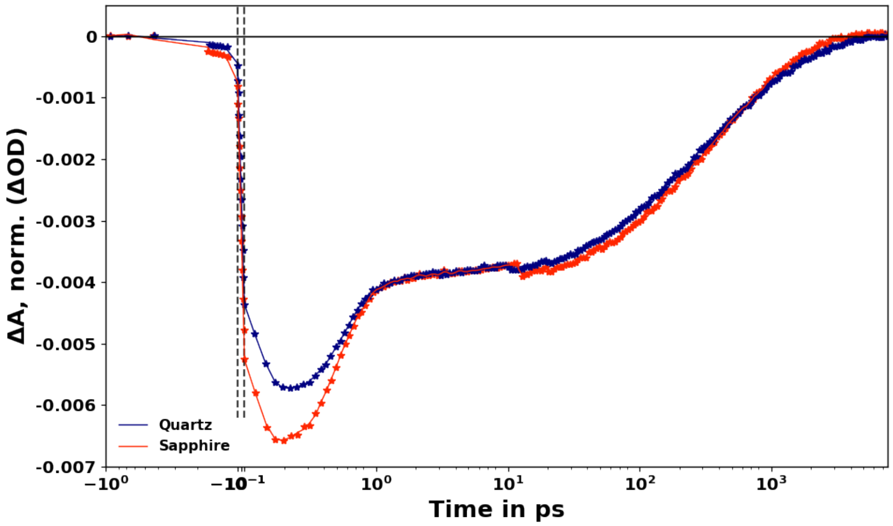

(b)

Figure S17: A comparison of 298 K transient absorption measured at the same power on two substrates with different thermal conductivity (quartz vs. sapphire). Quartz is less thermally conductive than sapphire.

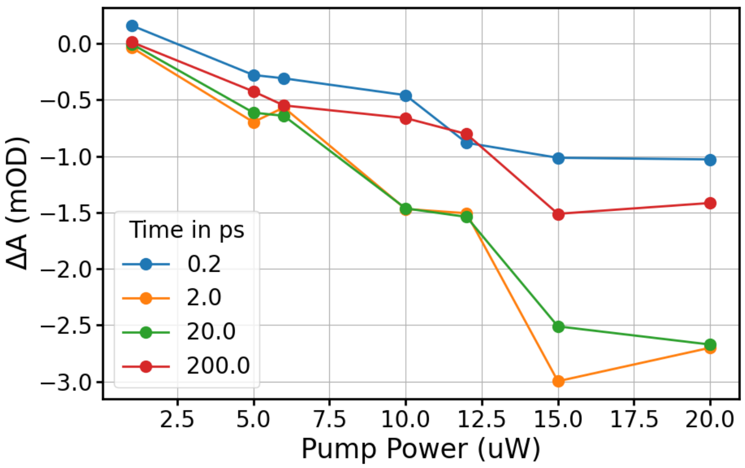

Figure S18: Optical density at 298 K in mOD at 718 nm (10 nm averaging window) as a function of pump power (at constant area) for a range of pump powers at different times. The linear regime appears to end either between 12 and 15 uW.

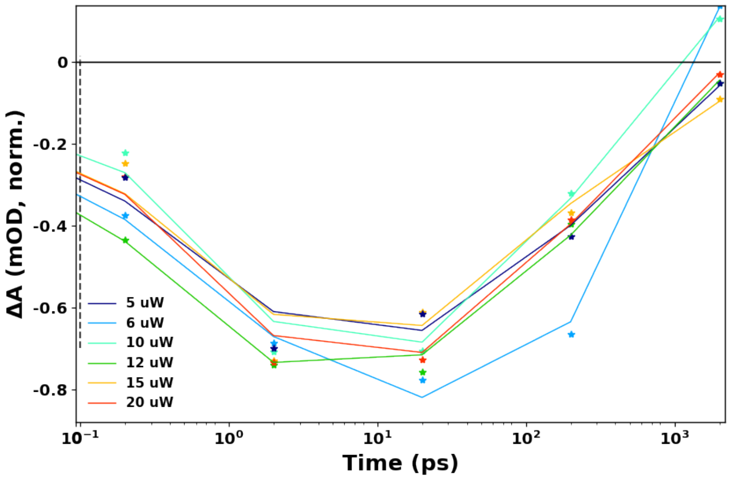

Figure S19: Normalized TA kinetics at 718 nm (10 nm averaging window) as a function of pump power at select times (298 K).

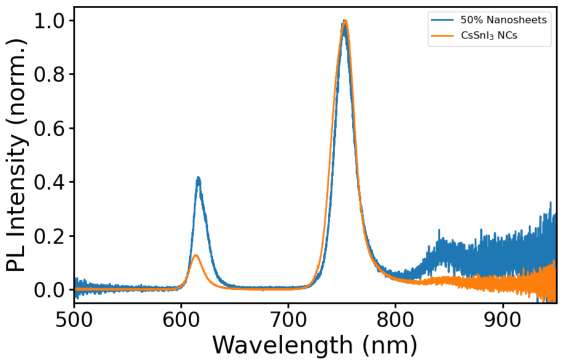

(a)

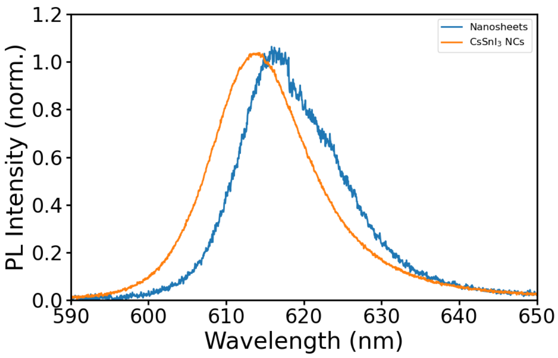

(b)

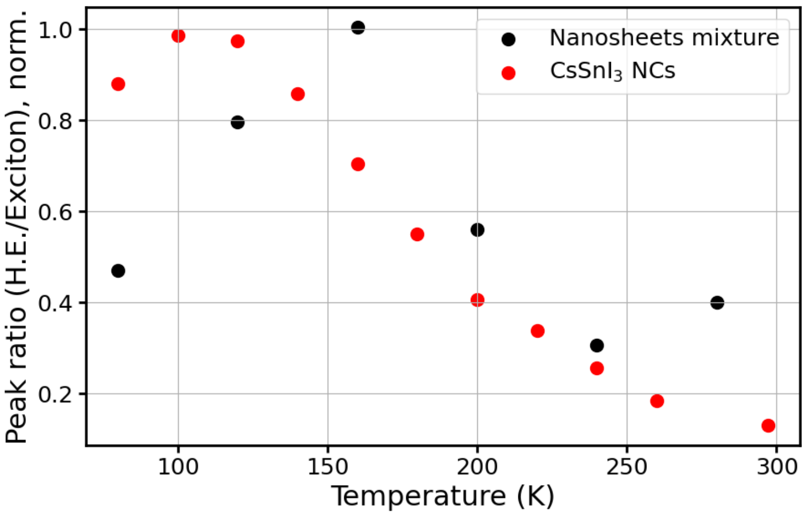

(c) Normalized ratio of integrated PL peak intensities.

Figure S20: Low-temperature photoluminescence at 120 K of a 50/50 mixture of 2D $[R{-}NH_3]_2SnI_4$ nanosheets and 3D $CsSnI_3$ NCs in a polystyrene matrix, measured under the same conditions as native $CsSnI_3$ samples.

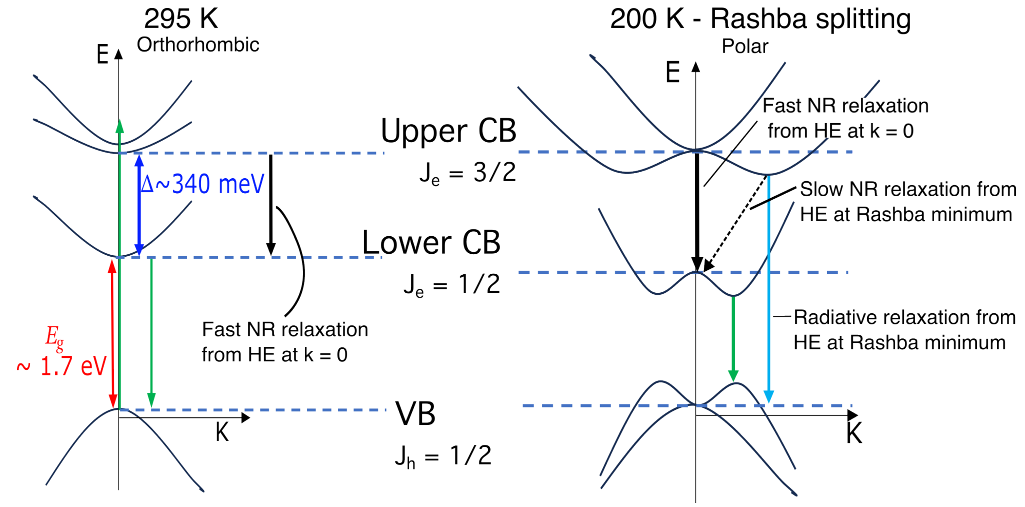

Figure S21: Scheme depicting energy band structure and kinetics behavior between 295 K and 200 K, showing hypothesized Rashba splitting of the states in the polar phase. The green downward arrows depict radiative relaxation of the exciton, which corresponds in the band picture to a transition from the $J_e = 1/2$ conduction band to the $J_h = 1/2$ valence band. Based conceptually on density function theory calculations of the bandstructure of ferroelectric $CsSnI_3$ by Swift & Lyons.
